# Supplementary material for: Formula Milk Supplementation and Bone Acquisition in 4–6 Years Chinese Children: A 12-Month Cluster-Randomized Controlled Trial
Source: Nutrients. 2023 Apr 21;15(8):2012. doi: 10.3390/nu15082012 (PMC10143222; doi:10.3390/nu15082012)
Supplement: Supplementary file 1 [file nutrients-15-02012-s001.zip › nutrients-2275433-supplementary.pdf]

Supplementary Tables S1–S3

Table S1. Composition of formula milk powder.

Nutrition Ingredients Category	Weight (per 100 g)	Nutrient Reference Values (NRV)%
Energy, kJ	1897	23%
Protein, g	19.0	32%
Fat, g	18.0	30%
Carbohydrates, g	52.0	17%
Dietary fiber (In terms of oligogalactose), g	3.0	12%
Sodium, mg	400	20%
Vitamin A, μgRE	430	54%
Vitamin D, μg	7.5	150%
Vitamin E, mg α-TE	2.30	16%
Vitamin K, μg	45.0	56%
Vitamin B_1, mg	0.75	54%
Vitamin B_2, mg	0.30	21%
Vitamin C, mg	30.0	30%
Niacin, mg	2.70	19%
Folic acid, μgDFE	140	35%
Pantothenic acid, mg	3.20	64%
Choline, mg	110.0	24%
Potassium, mg	350	18%
Magnesium, mg	30	10%
Calcium, mg	1200	150%
Iron, mg	8.1	54%
Zinc, mg	9.00	60%
Lutein, μg	150	–
Taurine, mg	30.0	–
Docosahexaenoic acid, mg	50.0	–
Arachidonic acid, mg	60.0	–
Lactoferrin, mg	30.0	–

Ingredients: Raw milk, demineralized whey powder, galactooligosaccharides, α-whey protein powder, milk salt, (3R, 3′r) -dihydroxy-β-carotene (≥118μg/100g), docosahexaenoic acid (DHA), arachidonic acid (ARA), yeast β-glucan, lactoferrin, taurine, lutein, bifidobacteria Bb-1 2(≥2×106CFU/g), Bifidobacterium lactis HN019(≥2×106CFU/g), and phospholipid. Vitamins: Vitamin A (retinyl acetate), vitamin D (cholecalciferol), vitamin E (DL-α-tocopherol acetate), vitamin B1(thiamine nitrate), vitamin K2, vitamin C (L-sodium ascorbate), folic acid, nicotinamide, D-calcium pantothenate, and choline chloride. Minerals: calcium carbonate, ferrous sulfate, and zinc sulfate.

Table S2. Comparison of means of bone mineral density (BMD) and bone mineral content (BMC) at baseline and their 12-month changes between the formula and control groups.

Variables	Control (n = 91)		Formula (n = 83)		Change (%)	*t*-test	ANCOVA*
	Mean	SD	Mean	SD	F v C		
Left forearm BMD, g/cm^2							
Baseline	0.179	0.030	0.182	0.029	1.33	0.576	0.568
6 m	0.185	0.030	0.193	0.030	4.37	0.065	0.061
12 m	0.197	0.029	0.212	0.030	7.80	<0.001	<0.001
Change_6 m	0.005	0.009	0.012	0.007	3.77	<0.001	<0.001
Change_12 m	0.019	0.014	0.031	0.012	6.66	<0.001	<0.001
Left forearm BMC, g							
Baseline	0.240	0.049	0.243	0.051	0.94	0.755	0.747
6 m	0.255	0.052	0.268	0.051	5.11	0.083	0.074
12 m	0.270	0.057	0.287	0.059	6.17	0.059	0.052
Change_6 m	0.015	0.019	0.026	0.018	4.55	<0.001	<0.001
Change_12 m	0.031	0.026	0.045	0.022	5.76	<0.001	<0.001
Left Calcaneus BMD, g/cm^2							
Baseline	0.182	0.039	0.186	0.039	1.72	0.581	0.536
6 m	0.194	0.034	0.196	0.038	0.80	0.770	0.756
12 m	0.214	0.032	0.217	0.036	1.11	0.643	0.611
Change_6 m	0.008	0.014	0.013	0.014	2.83	0.013	0.010
Change_12 m	0.029	0.021	0.033	0.019	2.02	0.231	0.203
Left Calcaneus BMC, g							
Baseline	0.194	0.038	0.199	0.038	2.34	0.410	0.354
6 m	0.206	0.033	0.207	0.037	0.73	0.769	0.755
12 m	0.226	0.031	0.228	0.035	1.04	0.640	0.608
Change_6 m	0.008	0.013	0.012	0.014	2.38	0.024	0.020
Change_12 m	0.028	0.021	0.031	0.018	1.64	0.286	0.258

*: Adjusting for corresponding baseline values (except for baseline measures analysis) depending on gender, age, mother's age, father's age, mother's BMI, father's BMI, mother's education level, father's education level, gestational age at birth, birth weight, breastfeeding time, mother's illness during pregnancy, use of nutritional supplements, dietary protein intake, dietary calcium intake, exercise, and sleeping time (method = stepwise).

Table S3. Comparison of means of bone metabolism markers at baseline and their 12-month changes between the formula and control groups.

Variables	Control (n = 83)		Formula (n = 83)		Change (%)	*t*-test	ANCOVA*
	Mean	SD	Mean	SD	F v C		
β-CTx, ng/ml							
Baseline	0.814	0.180	0.764	0.156	-6.17	0.051	0.052
6 m	0.989	0.210	0.909	0.185	-8.06	0.007	0.007
12 m	1.237	0.281	1.233	0.247	-0.32	0.924	0.922
Change_6 m	0.178	0.201	0.154	0.157	-3.04	0.375	0.392
Change_12 m	0.402	0.253	0.473	0.250	8.75	0.086	0.079
Trap-5b, pg/ml							
Baseline	676.5	151.5	676.8	156.0	0.05	0.990	0.990
6 m	470.7	136.9	422.3	132.2	-10.27	0.016	0.017
12 m	583.3	210.7	571.4	218.3	-2.04	0.720	0.724
Change_6 m	-204.1	202.9	-258.5	203.6	-8.04	0.085	0.087
Change_12 m	-95.7	270.6	-114.1	293.4	-2.73	0.688	0.687
Osteocalcin, ng/ml							
Baseline	57.32	12.31	57.55	12.22	0.40	0.903	0.899
6 m	64.53	13.56	61.57	14.54	-4.58	0.163	0.132
12 m	63.36	14.90	62.18	12.79	-1.86	0.592	0.580
Change_6 m	7.085	11.547	2.733	10.288	-7.59	0.012	0.017
Change_12 m	7.419	12.902	4.432	13.920	-5.21	0.181	0.173
BAP, μg/L							
Baseline	84.59	19.73	86.86	17.83	2.68	0.427	0.437
6 m	91.62	17.67	91.56	14.86	-0.06	0.982	0.981
12 m	93.23	19.41	94.21	14.81	1.05	0.727	0.727
Change_6 m	8.995	16.179	5.052	15.848	-4.66	0.117	0.111
Change_12 m	9.561	15.290	8.592	13.070	-1.15	0.683	0.673
ALP, U/L							
Baseline	220.0	56.7	232.1	44.9	5.50	0.104	0.099
6 m	283.6	60.9	282.7	53.1	-0.32	0.916	0.917
12 m	253.6	54.9	244.4	49.6	-3.61	0.262	0.242
Change_6 m	59.31	34.43	51.24	31.64	-3.67	0.102	0.110
Change_12 m	35.37	45.84	12.39	33.41	-10.44	<0.001	<0.001

25(OH)D, ng/ml							
Baseline	26.28	4.34	28.13	4.68	7.06	0.008	0.007
6 m	22.36	4.75	26.01	5.05	16.29	<0.001	<0.001
12 m	26.31	4.15	28.54	4.93	8.47	0.002	0.002
Change_6 m	-3.697	2.734	-2.241	2.817	5.54	0.001	0.001
Change_12 m	0.125	3.152	0.254	3.109	0.49	0.803	0.806
25(OH)D_2, ng/ml							
Baseline	2.327	0.550	2.326	0.515	-0.03	0.994	0.994
6 m	2.383	0.578	2.471	0.927	3.73	0.452	0.433
12 m	2.250	0.225	2.250	0.298	0.00	1.000	1.000
Change_6 m	0.025	0.684	0.085	0.922	2.57	0.649	0.634
Change_12 m	-0.057	0.800	-0.070	0.548	-0.55	0.909	0.907
25(OH)D_3, ng/ml							
Baseline	25.41	4.32	27.54	4.56	8.40	0.002	0.001
6 m	21.78	4.75	24.71	5.33	13.47	<0.001	<0.001
12 m	25.72	4.57	27.90	5.22	8.48	0.005	0.004
Change_6 m	-3.844	3.380	-2.358	3.503	5.85	0.007	0.007
Change_12 m	0.490	3.404	0.511	3.522	0.09	0.970	0.970
PTH, pmol/L							
Baseline	1.986	0.578	2.117	0.685	6.63	0.178	0.162
6 m	2.426	0.625	2.237	0.606	-7.80	0.043	0.042
12 m	2.751	0.829	2.601	0.666	-5.46	0.200	0.202
Change_6 m	0.504	0.592	0.202	0.687	-15.22	0.003	0.003
Change_12 m	0.710	0.703	0.500	0.685	-10.61	0.066	0.061
IGF-1, ng/ml							
Baseline	99.98	31.61	103.90	37.22	3.92	0.461	0.408
6 m	108.84	32.27	119.32	42.54	9.63	0.066	0.051
12 m	117.30	40.52	121.23	42.15	3.36	0.548	0.533
Change_6 m	8.52	22.92	16.88	19.35	8.36	0.014	0.018
Change_12 m	10.75	26.14	18.40	24.40	7.65	0.070	0.068

*: Adjusting for corresponding baseline values (except for baseline measures analysis) depending on gender, age, mother's age, father's age, mother's BMI, father's BMI, mother's education level, father's education level, gestational age at birth, birth weight, breastfeeding time, mother's illness during pregnancy, use of nutritional supplements, dietary protein intake, dietary calcium intake, exercise, and sleeping time (method = stepwise).

Abbreviations: β-CTx, β-C-terminal telopeptides; ALP, alkaline phosphatase; BAP, bone-specific alkaline phosphatase; Change_6 m/ Change_12 m: changes in BMD or BMC from baseline to 6/12 months; Change (%): percentage changes = (changes/baseline value) × 100%; IGF-1, insulin-like growth factor-1; PTH, parathyroid hormone.
